# Combining techniques for screening and evaluating interaction terms on high-dimensional time-to-event data

**DOI:** 10.1186/1471-2105-15-58

**Published:** 2014-02-26

**Authors:** Murat Sariyar, Isabell Hoffmann, Harald Binder

**Affiliations:** 1 Institute of Medical Biostatistics, Epidemiology and Informatics, Medical Center of the Johannes Gutenberg University, Mainz 55131, Germany; 2 Institute of Pathology, Charite – University Medicine Berlin, Campus Benjamin Franklin, Berlin 12200, Germany

**Keywords:** Boosting, High-dimensional data, Model selection, Model complexity, Prediction error curves, Random forest, Time to event settings

## Abstract

**Background:**

Molecular data, e.g. arising from microarray technology, is often used for predicting survival probabilities of patients. For multivariate risk prediction models on such high-dimensional data, there are established techniques that combine parameter estimation and variable selection. One big challenge is to incorporate interactions into such prediction models. In this feasibility study, we present building blocks for evaluating and incorporating interactions terms in high-dimensional time-to-event settings, especially for settings in which it is computationally too expensive to check all possible interactions.

**Results:**

We use a boosting technique for estimation of effects and the following building blocks for pre-selecting interactions: (1) resampling, (2) random forests and (3) orthogonalization as a data pre-processing step. In a simulation study, the strategy that uses all building blocks is able to detect true main effects and interactions with high sensitivity in different kinds of scenarios. The main challenge are interactions composed of variables that do not represent main effects, but our findings are also promising in this regard. Results on real world data illustrate that effect sizes of interactions frequently may not be large enough to improve prediction performance, even though the interactions are potentially of biological relevance.

**Conclusion:**

Screening interactions through random forests is feasible and useful, when one is interested in finding relevant two-way interactions. The other building blocks also contribute considerably to an enhanced pre-selection of interactions. We determined the limits of interaction detection in terms of necessary effect sizes. Our study emphasizes the importance of making full use of existing methods in addition to establishing new ones.

## Background

As more and more high-dimensional molecular data is amassed, the importance of biomarker research increases. Specifically, predictive biomarkers are usually wanted in order to predict risks associated with diseases. When building multivariate risk prediction models for finding such biomarkers, it is desirable to produce sparse models. The sparsity of the resulting models facilitates the biological and statistical interpretation [1-4]. Approaches such as componentwise boosting [5] or the LASSO [6-8] achieve sparsity by performing variable selection and parameter estimation simultaneously. There are two frequently occurring problems in this context: first, lack of reproducibility of variable selections across different studies, for example concerning gene expression data [9-11]; second, no established approaches to account for interactions. The latter deficit can lead to selection of wrong variables or biased parameter estimations. The first problem, i.e. the inability to confirm most of the published gene related signatures, has led to doubts whether signatures should be produced at all. However, the failure of finding stable signatures could to some extent be ascribed to inadequate modeling. Approaches that are more comprehensive are necessary, for example, combining molecular data with annotation and clinical information [9,12-15]. One ingredient should be to incorporate promising interactions in the model. Many tools for modeling interactions exist, but, as far as we know, no systematic investigations of potential building blocks are available.

Examples for promising modeling strategies that can account for interactions are penalized regression models [16,17], logic regression [18,19], multifactor-dimensionality reduction [20,21] or random forests [22,23]. For a comprehensive review regarding interaction (pre-) selection approaches, we refer to [24]. Logic regression and multifactor-dimensionality reduction are primarily destined for discrete marker data, e.g., for single nucleotide polymorphism data. In contrast to that, penalized or regularized regression models cover more general types of data. Their main property is to put a penalty on the model parameters, which correspond to marker effects, for estimation. The usage, for example, of an *L*_1_-penalization forces most of the estimated parameters to be zero, i.e., the values of the corresponding covariates do not influence predictions obtained from the fitted model. Even though these models are primarily used for main effect selections, there is an increasing interest in incorporating interactions [25-27]. When there is no a priori knowledge, such approaches either require the interactions to be formed by variables that represent main effects or that interaction terms are created by combining the covariates in a certain way, e.g., by producing all distinct two-way interactions (or by coarsening the input space before producing the interactions [28]). The first route can lead to false negatives even if the true interactions have relevant marginal effects, and the second one neglects the fact that it is frequently either not feasible or computationally too expensive to consider all possible interactions. Altogether, this means that a screening method for promising interaction terms is in most cases necessary, especially for higher order interactions. The potential of random forests to provide non-parametrical means for handling various kinds of interaction structures makes them attractive as an interaction screening method for penalized regression models. However, apart from some interesting theoretical results (see [29,30]) and positive empirical findings regarding prediction performances (e.g., [31-33]), the ability to extract information from random forests is considered problematic. The main objection is that established variable importance measures seem to be unable to detect relevant interaction effects in the absence of strong marginal components [34-36].

*Variable importance measures* (VIMs) for random forests are meant to extract the information contained in forests. Established VIMs are the *Gini*, the *permutation accuracy*, or the *minimal depth* importance (see [37-39]). The first measure uses the mean improvements in the Gini index in a forest related to the investigated variable. Permutation accuracy importance measures the change in prediction accuracy of the forest when the values of a variable are permutated randomly, and minimal depth importance is roughly related to the mean minimum distance (the depth) from the root node to the investigated variable. These measures can also be used for finding interactions in the forest. For example, the permutation accuracy importance can easily be extended such that the values of two variables are permutated randomly [37]. These variable importance measures lead to a ranking of variables, in which interaction information is assumed to enter in some way. Whether these interactions are statistically relevant can be evaluated by penalized regression models. Hence, a comprehensive evaluation can consist of two parts: extracting interaction terms based on random forest information and estimating a statistical regression model based on all available variables and identified interaction terms.

In this paper, we show building blocks for evaluating and incorporating interactions terms in high-dimensional time-to-event settings, in particular for settings in which it is very computationally expensive to check all possible interactions with an exhaustive search algorithm. The main ingredients are random survival forests (RSF), a specific adaptation of random forests to time-to-event settings, and an incremental stagewise forward regression technique, called CoxBoost [40-42]. CoxBoost is a boosting technique based on the Cox proportional hazards model and combines variable selection with model estimation. For this purpose, it uses a penalized version of the partial log-likelihood and applies componentwise boosting. We investigate the effect of a combination of these approaches, the additional contribution of resampling, and the advantage of a special data pre-processing step. This work is a feasibility study; hence, we are first of all interested in investigating how several components can contribute to the solution of the interaction finding problem. The specific choice of the investigated tools is justified by their specific properties; however, there are alternatives to our decisions (see above). We are interested in predicting risks within time-to-event settings and we use methods established in these settings. In this context, we rely on some assumptions, such as the proportional hazard assumption.

In the next section, we present details of CoxBoost and RSF together with corresponding VIMs. After presenting evaluation tools and our interaction detection strategy, we outline a simulation design for the evaluation. In the Results Section, the findings of the simulation study are shown, and we illustrate our approach on two real-world applications. Finally, we describe limitations of the study and summarize our findings in the Conclusion Section.

## Methods

Time-to-event or survival data for *n* investigated entities is typically given as a set of triples *z*_*i*_=(*t*_*i*_,*δ*_*i*_,*x*_*i*_),*i*=1,…,*n*. The first component is the observed time for each entity *i* and is given by *t*_*i*_= min(*T*_*i*_,*C*_*i*_), where *T*_*i*_ is the event time and *C*_*i*_ is the censoring time from which on the entity is no longer observed. The second component is the event indicator *δ*_*i*_, which takes the value 1 if an event has occurred at the observed time (*T*_*i*_≤*C*_*i*_) and 0 if the event time is censored (*T*_*i*_>*C*_*i*_). The third element, *x*_*i*_, is the vector of values of the *p* covariates observed at baseline.

### CoxBoost

As a forward stagewise regression technique in the time-to-event setting, we use a likelihood-based boosting variant, called CoxBoost [15,43]. This technique is based on the Cox proportional hazards model, which relates the hazard *λ*(*t*|**x**_*i*_), i.e. the instantaneous risk of having an event at time *t*, given the covariate information in *x*_*i*_, for entity *i*, in the following way:

$$\lambda (t|x_{i})=\lambda_{0}(t)\exp(\overset{T}{\underset{i}{x}}\beta ),$$

where the baseline hazard *λ*_0_(*t*) is left unspecified. Usually, the parameter vector *β*=(*β*_1_,…,*β*_*p*_)^*T*^ is estimated by maximizing the partial log-likelihood (PLL):

$$\;\text{PLL}(\beta |x_{1},\ldots ,x_{n})\;=\;\overset{n}{\underset{i=1}{\sum }}\;\delta_{i}\;\;\left(\;x_{i}^{T}\beta -\;\log\;\left(\overset{n}{\underset{j=1}{\sum }}\;I(t_{i}\leq t_{j})\;\exp(x_{j}^{T}\;\beta )\;\right)\;\right)$$

with indicator function *I* (see also [44]). However, such a procedure is not feasible for *p*>*n*. Therefore, CoxBoost uses a penalized version of the PLL and applies componentwise likelihood-based boosting [40,41,45]. Conventional CoxBoost starts with parameter estimates ${\hat{\beta }}^{\left(0\right)}={\left(0,\ldots ,0\right)}^{T}$. In each boosting step *k*=1,…,*B*, only one coefficient is updated. In order to determine which component *j*^∗^ should be updated in step *k*, the penalized univariate PLL with argument $\theta_{j}^{\left(k\right)}$, *j*∈{1,…,*p*}, is considered:

$${\text{PLL}}_{\text{pen}}\left(\theta_{j}^{\left(k\right)}\right)=\text{PLL}\left(\theta_{j}^{\left(k\right)}\right)-\frac{\rho }{2}{\left(\theta_{j}^{\left(k\right)}\right)}^{2},$$

with fixed penalty parameter *ρ*>0 and the variable parameter $\theta_{j}^{\left(k\right)}$. In $\text{PLL}\left(\theta_{j}^{\left(k\right)}\right)$, all parameter components with indices unequal to *j* are set to the corresponding components of ${\hat{\beta }}^{\left(k-1\right)}$. The parameter vector component *j*^∗^ is the one that leads to the maximum value of ${\text{PLL}}_{\text{pen}}\left(\theta_{j}^{\left(k\right)}\right)$. Instead of maximizing the penalized PLL for each candidate *j*, using the standard Newton-Raphson algorithm, the penalized score statistic can be used as a criterion

$$U_{j}^{\left(k\right)}{\left(I_{j}^{\left(k\right)}+\rho \right)}^{-1}U_{j}^{\left(k\right)},$$

where $U_{j}^{\left(k\right)}$ is the value of the score function *U*(*θ*)=*∂*PLL(*θ*)/*∂θ* for $\theta \;=\;\theta_{j}^{\left(k\right)}\;=\;0$, and $I_{j}^{\left(k\right)}$ is the value of the Fisher information *I*(*θ*)=*∂*^2^PLL(*θ*)/*∂θ*^2^, again for $\theta =\theta_{j}^{\left(k\right)}=0$. The covariate *j*^∗^ with the largest value of the score statistic is selected for an update of the form:

$${\hat{\beta }}_{j^{*}}^{\left(k\right)}={\hat{\beta }}_{j^{*}}^{\left(k-1\right)}+{\hat{\theta }}_{j^{*}}^{\left(k\right)}$$

while ${\hat{\beta }}_{j}^{\left(k\right)}={\hat{\beta }}_{j}^{\left(k-1\right)}$ for all covariates *j*≠*j*^∗^. The tuning parameter *ρ* is typically set to $\underset{i}{\sum }\delta_{i}\cdot (\frac{1}{\nu }-1)$, with *ν*∈(0,1] as the relative step size factor. The number of boosting update steps can be determined by a cross-validation procedure.

One salient feature of this forward stagewise regression technique is that it inherently avoids ’breaking up a large main effect coefficient into a sum of smaller pieces’ in contrast to, for example, non-boosted regression models with *L*_2_-penalization (see [16]). In addition to that, CoxBoost has many extensions. It is, for example, possible to force the inclusion of a number of covariates into the model by suspending penalization for them [15]. This is relevant for settings with few clinical covariates and a large number of molecular variables. In this case, the coefficient estimates of the mandatory covariates are updated before the other covariates. Further, more than one coefficient can be updated in each boosting step, or the penalization parameter can vary from step to step. CoxBoost and all these features are implemented as an R-package, correspondingly called CoxBoost[46].

### Random forests

Random forests are ensembles of – usually binary – classification or regression trees [22]. Usually unpruned trees are generated based on resamples of the original data and a random component in the splitting procedure, which implies that in every knot splitting is based on the number *mtry* of randomly selected variables. Unpruned trees in the context of random forests are rather unproblematic in terms of overfitting on training data; however, they can have detriment effects on the consistency of the response estimations [29,47]. Each path in such generated trees represents a sequence of splits that leads to the response of cases corresponding to that path. The final model response is determined by aggregation, e.g. averaging the responses of a case over all trees.

Random forests can detect and deal with small effects, interactions and non-linear associations, making no assumptions about the corresponding functional form [48]. All of these characteristics are also valid for trees. However, one important rationale behind random forests is the de-correlation of information that is represented in single trees, which reduces the corresponding variances – a bagging phenomenon [30,49] – and the grouping property of trees. The latter property relates to the fact that a split on a variable from a cluster of correlated variables is frequently followed by splits of other members of that group [50]. A further advantage of forests over trees is that they can approximate smooth functions without the necessity of having a large number of leaves in a tree, due to the smoothing effect of the bagging phenomenon [51]. Random forests perform relatively well off the shelf [52] with the default-values for *mtry* (=) and for the number of trees in a forest (=1000).

As one specific adaptation of random forests to right-censored time-to-event data, we consider random survival forests (RSF) [53]. For a – computationally expensive – alternative, see party. The response for RSF is the cumulative hazard function (CHF), defining an ensemble predicted value with respect to ’mortality’. For splitting, typically the Logrank test is used [54]. Hence, the homogeneity of nodes in the tree is a result of maximizing the difference of event probabilities between daughter nodes. For each entity in the data set, the ensemble CHF is calculated by averaging the Nelson-Aalen estimator of all leaves, into which the entity drops [53,55]. For a terminal node *h* with *N*(*h*) distinct event times *t*_1,*h*_<*t*_2,*h*_<…<*t*_*N*(*h*),*h*_, this estimator is given as

$${Ĥ}_{h}(t)=\underset{l:t_{l,h}\leq t}{\sum }\frac{d_{l,h}}{Y_{l,h}},$$

where *d*_*l*,*h*_ and *Y*_*l*,*h*_ are the number of deaths and entities at risks at time *t*_*l*,*h*_. RSF are implemented in the R-package randomSurvivalForest[56].

### Variable importance measures for random forests

Various variable importance measures (VIMs) can be used for selecting variables. There are two well-known VIMs: *Gini importance* and *permutation accuracy importance* (PAM). Another VIM is the mean minimal-depth measure, which has been proposed recently. Roughly, it measures the shortest distance (depth) from the root node to the parent node of the maximal subtree (the largest subtree whose root node splits with respect to the variable investigated). For further details, we refer to [50]. Different VIMs can produce different rankings; for example, the Gini importance was found to be highly affected by selection bias, e.g., continuous variables are preferred to categorical variables with only few categories [38]. In the following, we focus on PAM, because it is widely accepted and relates to the concept of simulating a null distribution (necessary for computing p-values), even though we are aware of potential problems [50,57]. For further information regarding VIMs, we refer to [58] and [59].

There are two versions of PAM. In its common version it is computed with respect to random permutations of the components of *x*_*j*_=(*x*_1*j*_,…,*x*_*nj*_)^*T*^, which breaks the association of *x*_*j*_ with the response and all variables. In a more sophisticated variant, which is unique to RSF with respect to survival data [50], the vector *x*_*i*_=(*x*_*i*1_,…,*x*_*ip*_) related to entity *i* is dropped down in all trees, in which it was out of bag in the training process; whenever a split node for an investigated variable is encountered, the corresponding vector *x*_*i*_ is randomly assigned to one of the daughter nodes. In both variants, the variable importance results from the prediction error of the altered forest minus the prediction error of the non-altered forest. The larger the importance values of a variable, the higher its value for prediction. It is important to notice that PAM is tied to the error measure used. One frequently used error measure for RSF, which we use here as well, is Harrell’s concordance index, which measures the discrimination ability of a model [60].

### Tools for finding effects in time-to-event data

In high-dimensional settings, the problems of extracting relevant information by regression models are aggravated compared to the low-dimensional counterparts. For example, even if stepwise regression introduces biases related to multiple test problems (see, for example, [61,62]), it nevertheless provides a means for tackling variable selection issues in a comprehensive manner. It is therefore crucial to investigate mechanisms and measures for an adequate model selection on high-dimensional data. Three issues have to be addressed simultaneously: (1) a sparse variable selection, (2) representing the relevant structure in the data, and (3) good prediction performance. We try to tackle these issues and in particular concentrate on integrating substantial interactions into the model.

The likelihood-based boosting algorithm promises sparse and stable variable selection, which is a consequence of simultaneous selection and estimation in a multivariable model. Naturally, variable selection stability also depends on the quality of the data (see, for example, [63]), and for obtaining high-quality molecular data frequently appropriate pre-processing steps are necessary, e.g., background correction and normalization. Concerning the other two issues (representing the relevant structure in the data and good prediction performance) Yang [64] strikingly demonstrates that best predictive models usually contain irrelevant features and important features often do not lead to best prediction performances (see also [65,66]). Whenever we encounter the trade-off between relevance and usefulness for prediction, we prioritize ’finding relevant variables’ over prediction performance.

The models are evaluated within a resample procedure for estimating sensitivity and stability. As a performance measure adapted for time-to-event endpoints, we use the Brier score [67,68]. The Brier score is a strictly proper scoring rule, i.e. it is optimal only at the true probability model (see [69]). For example, the area under the curve (AUC) is not a strictly proper rule, because it can lead to optimal values for different probability models (slight changes of probabilities often do not matter). Two common resampling techniques are cross-validation (CV) and bootstrapping. Cross-validation partitions the data into folds and evaluates prediction performance on every single fold with models fitted to the data from the remaining folds; a more precise characterization for CV is therefore ’subsample technique’. Both techniques can cause problems (see [38,70]), and we decided to use subsampling with splits of relative size 0.632 to (1-0.632), because this seems to work well in many settings [71,72]. Such a subsampling procedure is roughly comparable to a 3-fold CV (see [73,74]).

The Brier score quantifies the squared deviation between predicted survival probability and observed survival status and is independent from the assumed survival model. When ${Ĥ}_{0}$ is the estimated cumulative baseline hazard at baseline and $\hat{\beta }$ denotes the estimated coefficients, the predicted survival probability is given by

$$\hat{\pi }(t,x)=1-\exp\left(-{Ĥ}_{0}(t)\exp(x^{T}\hat{\beta })\right)$$

and the expected Brier score tracked over time (i.e., the expected prediction error curve) has the form

$$\text{Err}(t;\hat{\pi }):=E_{X}\left[{\left(\delta (t)-\hat{\pi }(t,x)\right)}^{2}\right],$$

where *δ*(*t*) is the true survival status at time *t*. Typically the survival status at time *t* will be right censored for some observations. Thus, inverse probability of censoring weights (IPCW) were proposed to avoid the related bias [68,75]. The IPCW for individual *i* is defined as

$$W_{i}(t;\hat{P})=\frac{I(t_{i}\leq t)\delta_{i}(t)}{\hat{P}(t_{i}-|x_{i})}+\frac{I(t_{i}>t)}{\hat{P}(t|x_{i})},$$

where $\hat{P}(s|x_{i})$ is a consistent estimate of probability that the censoring time is larger than *s*, given *x*_*i*_. *I*(·) is again the indicator function. The cross-validation estimate of the Brier score tracked over time is then

$$\;{\hat{\text{Err}}}_{\text{boot}}(t;\hat{\pi }):=\frac{1}{B}\overset{B}{\underset{b=1}{\sum }}\frac{1}{\left|I\setminus I_{b}\right|}\underset{i\notin I_{b}}{\sum }{\left(\delta_{i}(t)-{\hat{\pi }}_{b}(t,x_{i})\right)}^{2}W_{i}(t;\hat{P}).$$

Here, *B* is the number of resamples, *n* the number of rows, and $I_{b}$ the indices of those cases that are included in the resample *b*.

### Assembling of building blocks into an interaction detection strategy

Our comprehensive strategy consists of three parts: (a) first main effect detection, (b) pre-selection of interactions terms,(c) final model selection. Parts (a) and (c) use CoxBoost and are fixed. Here, we rely on the ability of CoxBoost to produce sparse models and to include important variables. In part (b), we consider the following building blocks: (BB1) subsampling, (BB2) random forests, and (BB3) orthogonalization as a data pre-processing step. Different decisions concerning the building blocks lead to flexibility in part (b). When combining building blocks into comprehensive strategies, over-fitting to the data at hand and over-optimism could occur [76]. One way to account for that – besides the usage of independent validation data sets – is to evaluate the contribution of the building blocks to the results.

The use of an outer subsampling for interaction finding has the aim of enhancing the credibility of interaction information. Specifically, we use the variable inclusion frequency (VIFs), i.e. the proportion of times that the variable appeared in the model, for assessing the relevance of an interaction term. For example, when using random forests, this means that the number of random forests in which interaction terms are deemed relevant is the basis for a pre-selection of interactions. Here, an interaction term is assessed as relevant if both underlying variables have PAM values larger than zero in a random forest (typically, there are many variables with PAM values ≤0). In other words, variables have to be simultaneously important for a random forest.

When all building blocks are used for the pre-selection of interactions terms, random forests are applied to the data in a subsampling context and orthogonalization is used as a data-pre-processing step. Orthogonalization means that all variables not considered as main effects are made orthogonal to those that are indicated as main effects by CoxBoost in the first step. This leads to disentanglement of information, which might allow to determine variables and related interactions that contain information that was originally masked by main effects (a similar idea is employed in [27]). The strategy using all building blocks is described by the following pseudo-algorithm (rsf-VIF-res): 

1. Specify: Indices  of clinical covariates or other known main effect variables, number *S* of subsamples for pre-selecting interactions, and number *R* of pre-selected interaction terms. In case of identical VIF values for the *R*th and (*R*+1)th found interaction, all interactions with that VIF value are included as well.

2. Subsample the original data set *Z* in relation 0.632 to (1-0.632), leading to the data sets *Z*_*b*_ and $Z_{b^{\prime }}$. 

(a) **First pass main effects detection**: Run CoxBoost on *Z*_*b*_, possibly incorporating clinical covariates $\left\{x_{k}|k\in K\right\}$ without penalization. This leads to the model *CoxBoostM* and a list of main effects, given by the index set . Main effects and clinical covariates are used for orthogonalization (if this pre-processing step is considered) in the pre-selection step and as unpenalized variables in the final CoxBoost model.

(b) **Pre-selection of interaction terms**: If ℳ∪K is non-empty, regress all covariates with indices {1,…,p}∖(ℳ∪K) on the variables in (ℳ∪K). Subsequently, compute the corresponding residuals of the covariates, which leads to the data matrix ${\tilde{Z}}_{b}$ (building block (BB3)). Subsample *S* times data from ${\tilde{Z}}_{b}$ – from *Z*_*b*_, when  is empty – in relation of 0.632 to (1-0.632) and generate RSF on each larger subsample (building blocks (BB1) and (BB2)). Construct interaction terms by all pairs of variables with PAM values greater 0 on every subsample and compute VIFs of the interactions terms at the end of the subsampling process. Select the *R* most frequent pairs.

(c) **Final model**: covariates are $x_{k},k\in K$, $x_{i},i\in ℳ$, and the *R* selected cross product terms of (b). Run CoxBoost on *Z*_*b*_ with these covariates without penalization for covariates with indices in , leading to model *cb*_fin_.

(d) **Compute prediction error**: Apply *cb*_fin_ on $Z_{b}^{\prime }$ and compute the Brier score.

For assessing the contribution of building blocks, we successively remove one of them in the pre-selection step, leading to following alternatives to step (b): 

b1 Do the same as in rsf-VIF-res, but without orthogonalization. (rsf-VIF)

b2 Replace rsf in rsf-VIF by CoxBoost: subsample *S* times data from *Z*_*b*_ in relation 0.632 to (1-0.632) and run CoxBoost on each of the larger data sets. Finally, compute VIFs related to the variables selected by CoxBoost in each subsample, and create *R* pairs, i.e. interaction terms, related to the variables with the highest VIFs. (cb-VIF)

b3 Omit subsampling in cb-VIF: compute all distinct cross product terms of covariates with indices in . Here, *S* is superfluous and *R* is not needed, if $|\sqrt{\left|ℳ\right|}|\leq R$; otherwise, select randomly *R* interactions from all cross product terms. (cb-crossp)

There are many more alternatives, which are not considered due to a limited space and for reasons of clarity.

### Simulation design

For a systematic analysis of the building blocks and the corresponding interaction detection strategies, a time-to-event simulation study was conducted. Here, we define interactions as effects based on multiplicative combinations of variables. The main interest concerns the ability of the strategies to find relevant interactions and especially those that might be difficult to detect, i.e., variables in interactions are not members of the set of true main effects. The secondary focus is on the prediction performance, which highly depends on the effect sizes of main effect variables and interaction terms.

The simulation scenarios are designed to mimic simple yet realistic settings, e.g. microarray studies. We simulate independent as well as correlated data for a time-to-event end point. Table 1 summarizes the scenarios and shows the effect sizes of the main effects and interactions. The number of covariates is fixed as 1000 (=*p*), the sample size is fixed as 150 (=*n*), and all covariates are from a standard normal distribution (except for Sim22_bin with 4 binary variables and the scenarios with correlated data). The covariates not indicated in the table have zero effect sizes. For each simulation scenario, 50 datasets are generated. Survival times and censoring times are generated from an exponential distribution with baseline hazard $\lambda =\frac{1}{20}$ (see also [77]).

**Table 1:** The effect sizes of non-zero effects in each scenario

**Scenarios:**	**Effect size ME**	**Effect size Int**	**Corr value**	**Block**
				**size**
Sim42	(3, 3, -3, -3)	(5,-5)		
Sim22_1.0	(0.9, -0.9)	(1.0, -1.0)		
Sim22_0.5	(0.9, -0.9)	(0.5, -0.5)		
Sim22_0.25	(0.9, -0.9)	(0.25, -0.25)		
Sim22_1.5	(0.9, -0.9)	(1.5, -1.5)		
Sim22_2.0	(0.9, -0.9)	(2.0, -2.0)		
Sim22_2.5	(0.9, -0.9)	(2.5, -2.5)		
Sim22_bin	(0.9, -0.9)	(1.0, -1.0)		
Sim22_corr01	(0.9, -0.9)	(1.0, -1.0)	0.1	5
Sim22_corr03	(0.9, -0.9)	(1.0, -1.0)	0.3	5
Sim22_corr05	(0.9, -0.9)	(1.0, -1.0)	0.5	5
Sim22_corr07	(0.9, -0.9)	(1.0, -1.0)	0.7	5

ME: main effect. Int: interaction. Corr: correlation. In all scenarios, the samples size is 150 (=*n*) and the number of covariates is 1000 (=*p*). The effect sizes are given in the form ‘(coefficient value of effect 1, coefficient value of effect 2)’. For the scenarios with correlations, *p* divided by the block size (200) gives the dimension of the normal distribution from which the values of the variables in a block are sampled, and the correlation value is the value at the off-diagonals of the corresponding covariance matrix. In scenarios Sim42 and Sim22_1.0, all interaction detection strategies are covered. All other scenarios are used for investigating rsf-VIF-res.

Sim42 refers to the simulation case with 4 main effects and 2 interactions that are composed of the main effects and Sim22_*x* to the cases with 2 main effects and 2 interactions that are not related to these main effects; in other words: they are composed of variables that have zero effect sizes. If *x* is numeric, it gives the uniform effect size; *x*= "bin" denotes the case of interactions composed of binary variables, and *x* beginning with "corr" relates to cases with variables that are block-correlated with a uniform correlation coefficient *c*, *c*∈{0.1,0.3,0.5,0.7}, across the 200 fiver-blocks, i.e., values of variables are sampled from a 5-dimensional normal distribution with the same variance matrix (the same correlation value at the off-diagonals and variance of 1) over all blocks. Here, main effects and variables in interactions terms stem from different blocks.

In scenarios Sim42 and Sim22_1.0, all interaction detection strategies are considered for evaluating the effect of the building blocks. The other scenarios are used to investigate the behavior and the limits of rsf-VIF-res. The simple scenario Sim42 is used for ascertaining that the strategies are capable of finding the relevant main effects. Scenario Sim22_1.0 is the reference scenario for the scenarios with non-smooth interactions, i.e. interactions incorporating binary covariates, and correlated variables.

The performance of a strategy is measured by the number of correct non-zero variables in the models, i.e. the variable selection sensitivity with respect to the main effects and the interaction terms, and by the prediction error (Brier score). Specificity values or predictive values are not separately listed in the result tables. However, these measures can be deduced from the sensitivity values and the number of selected variables.

In order to obtain one interpretable measure for the prediction performance, the Brier scores tracked over time are aggregated by computing the integrated prediction error curves (IPECs) for each model. Furthermore, the IPECs of the estimated models (${\text{IPEC}}_{S_{i}}$) are considered relative to the IPEC of the corresponding Kaplan-Meier estimator (IPEC_KM_):

$${\text{rIPEC}}_{i}:=\frac{{\text{IPEC}}_{\text{KM}}-{\text{IPEC}}_{S_{i}}}{{\text{IPEC}}_{\text{KM}}}.$$

In other words, rIPEC gives the relative improvement of prediction performance of strategy *S*_*i*_ compared to the prediction performance of the Kaplan-Meier.

## Results

### Simulation study

The simulation was conducted in R-3.0.2 with following main settings for the model implementations used in our strategies. Parameters not listed are considered secondary and were set to their default values:

**CoxBoostpenalty**: (Number of events) $\cdot (\frac{1}{0.05}-1)$. Penalty value for the updates in each boosting step. **standardize**: TRUE. Covariates are standardized. **stepno**: As computed by cv.CoxBoost. Number of boosting steps.

**RSFmtry**: Square root of the number of variables (default value). **ntree**: 1000. Number of trees grown (default value).

The parameter values of the strategies described in the Methods Section were chosen in the following way: no clinical covariates, hence K={}; the number of subsamples (*S*) for pre-selecting interactions was 50, and the number of pre-selected interaction terms (*R*) was 10000. For all scenarios, data were are randomly generated 50 times. The results for scenarios Sim42 and Sim22_1.0 are given in Table 2. The relevant columns are: the number of selected interactions by the corresponding screening method (IntScreen), the number of total variables in the final model (VarsTotal), the sensitivity with respect to the inclusion of true main effects (MainSensi), the sensitivity with respect to the availability of true interactions from the screening step (IntSensiA), the sensitivity with respect to the inclusion of true interactions in the final model (IntSensi), and the rIPEC values of CoxBoostM and the final model. For simplifying the discussion of the results, we will abbreviate the phrase ‘random forests together with PAM’ by ‘random forests’ or ‘RSF’.

**Table 2:** Results of the simulation study for all strategies in scenarios Sim42 and Sim22_1.0

	**IntScreen**	**IntSensiA**	**VarsTotal**	**MainSensi**	**IntSensi**	**rIPEC**	
						**CoxBoostM**	**Final Model**
**Scenario Sim42**							
(cb-crossp)	213.78	0.7 (0.05)	14.3 (6.29)	0.845 (0.04)	0.7 (0.05)	0.12 (0.12)	0.4 (0.19)
(cb-VIF)	1573.06	0.88 (0.03)	19.42 (7.89)	0.845 (0.04)	0.88 (0.03)	|	0.43 (0.15)
(rsf-VIF)	24557.74	0.95 (0.02)	34.74 (7.97)	0.82 (0.04)	0.94 (0.02)	|	0.37 (0.15)
(rsf-VIF-res)	25740.32	0.97 (0.02)	32.02 (6.56)	0.845(0.05)	0.97 (0.02)	0.12 (0.122)	0.4 (0.12)
**Scenario Sim22_1.0**							
(cb-crossp)	89.56	0 (0)	17.28 (8.18)	0.95 (0.02)	0 (0)	0.12 (0.09)	0.09 (0.13)
(cb-VIF)	2058.5	0 (0)	19.12 (12.19)	0.87 (0.03)	0 (0)	|	0.09 (0.1)
(rsf-VIF)	17511.96	0.07 (0.01)	12.64 (10.85)	0.73 (0.07)	0.06 (0.02)	|	0.08 (0.08)
(rsf-VIF-res)	17701.72	0.4 (0.05)	19.9 (12.93)	0.81(0.04)	0.39(0.05)	0.12 (0.09)	0.14 (0.14)

IntScreen (given as mean) is the number of selected interactions by the corresponding screening method; IntSensiA (given as ‘sensitivity value (sd)’) is the sensitivity related to the availability of true interactions; VarsTotal (given as ‘mean (sd)’) is the number of total variables in the final model; MainSensi (given as ‘sensitivity value (sd)’) is the sensitivity related to the inclusion of true main effects; and IntSensi (given as ‘sensitivity value (sd)’) is the sensitivity related to the inclusion of true interactions. The rIPEC values (given as ‘mean (sd)’) are shown for CoxBoostM and the final model. The scenarios were repeated 50 times. Additional file 1: Figure S1 provides boxplots for further insights into the nature of the variability in rIPEC.

In scenario Sim42, use of random forests generate models with more than 30 variables in the mean (about twice the number seen Sim22_1.0), whereas the other two strategies result in less than 20 variables on average. This means that there are many false positive findings when using RSF, which has a negative impact on the rIPEC compared to the cb-strategies. On the other hand, rsf-VIF-res leads to the largest sensitivity values for main effects and interactions. Hence, there is a trade-off between sensitivity and prediction performance. For all pre-selection variants it seem that when true interaction are pre-selected (see IntSensiA), then almost all of them are selected in the final model. Comparing cb-crossp with cb-VIF, we see that subsampling can increase IntSensi without decreasing MainSensi, and this leads to the best rIPEC value in scenario Sim42. Use of random forest instead of CoxBoost for interaction pre-selection (rsf-VIF) increases IntSensi but leads to a slight reduction of MainSensi. The MainSensi and IntSensi values of rsf-VIF-res indicate that orthogonalization is not only important for further increasing IntSensi but also for a higher MainSensi value compared to rsf-VIF. Overall, in this scenario, subsampling is important and random forests should be applied on orthogonalized data for achieving the largest sensitivity values but even then, prediction performance cannot be improved compared to interaction pre-selection with CoxBoost.

Scenario Sim22_1.0 exhibits some differences to Sim42. First, all strategies lead to similar and moderate numbers of total variables in the final model. Hence, high IntScreen values in RSF strategies do not result in more false positives than interaction pre-selection variants that use CoxBoost. CoxBoost is not able to pre-select true interactions, with or without subsampling. Subsampling even reduces MainSensi values. Use of random forest further decreases MainSensi with a little compensation of increased IntSensi but at an interchange rate that makes a further reduction of rIPEC possible. Again, RSF has to be applied to the pre-processed data (rsf-VIF-res) for increasing MainSensi and IntSensi compared to rsf-VIF. Now, the increase in IntSensi is drastic, which leads to the best rIPEC value in this scenario. The IntSensi value is still moderate; however, one should bear in mind that the interactions are built by variables that do not represent main effects. This might be particularly relevant for real world applications: even a moderate variable inclusion frequency of an interaction term could indicate an important interaction if the underlying variables are irrelevant as main effects.

Both scenarios show that all building blocks are important, and in particular orthogonalization is important before applying RSF, i.e. disentangling information beforehand is crucial for pre-selecting interactions. CoxBoost is unlikely to benefit from such a pre-processing because it already applies some sort of orthogonalization during fitting (further experiments also point in that direction; data not shown).

That IntSensiA is often similar to IntSensi in both scenarios means that one can rely on the ability of CoxBoost to choose the right interaction terms out of those presented, regardless of IntScreen. Hence, it seems that the parameter *R* (number of selected interactions) can be quite high. For assessing the effect of *R*, we additionally investigated the same scenarios rsf-sVIF-res with *R*=1000 (see Additional file 1: Table S1). With this reduced *R*-value, sensitivities, VarsTotal, and rIPEC decreased; the latter two measures were in particular reduced in scenario Sim22_1.0. Thus, in case of doubt, *R* should be set to a larger value.

We also investigated the behavior of the parameter estimates of the main effects and the interaction terms. In no case did a true effect receive a wrong sign. In the mean, shrinkage of the coefficients was stronger in scenario Sim42 than in Sim22_1.0. This has two reasons: higher absolute values of the true coefficients and increased number of non-zero coefficients. As the results show, this increased shrinkage is not relevant for the sensitivity of the detection strategies. One can try to reduce shrinkage by reducing the value of the penalty parameter or manually increasing the number of step sizes; however, we would not recommend such intervention in a high-dimensional setting without good reasons (see below).

Table 3 shows the results for rsf-VIF-res in scenarios Sim22_0.25 - Sim22_2.5 and Sim_bin. The former scenarios show that with reduced effects of the interaction terms MainSensi increases. In other words, the final model ceases to find true main effects, if effect sizes of interactions terms are larger than the effect size of the main effects. In scenarios Sim22_*e*, *e*≥1.0, the rIPEC values are larger than that of CoxBoostM due to an increase both of MainSensi and IntSensi. Reducing the effect sizes of interactions below 1.0 seems to make them unimportant for the prediction performance of the final model. This leads to a further increase of MainSensi values and decreasing IntSensi values, causing rIPEC values of CoxBoostM to be larger than those of the final model. Nevertheless, InterSensiA has the largest value in Sim22_0.25, which means that random forests were frequently able to preselect the true interactions, even if the effect sizes of the interaction terms are small. Scenario Sim_bin exhibit another interesting feature: IntSensiA is larger and IntSensi smaller compared to scenario Sim22_1.0. This means that non-smooth interactions are found slightly better by the random forest, and yet CoxBoost was not able to select them all. The large sum of both sensitivity MainSensi and IntSensi values does not lead to an improvement of rIPEC compared to CoxBoostM, which probably is a result of a larger number of false positives in the final model.

**Table 3:** **Results of the simulation study for **rsf-VIF-res** in scenarios Sim22_0.25 - Sim22_2.5 and Sim_bin**

**Scenario**	**IntScreen**	**IntSensiA**	**VarsTotal**	**MainSensi**	**IntSensi**	**rIPEC**	
						**CoxBoostM**	**Final Model**
**Strategy rsf-VIF-res**							
Sim22_2.5	15231.62	0.3 (0.05)	16.04 (14.72)	0.29 (0.05)	0.3 (0.05)	0 (0.06)	0.1 (0.16)
Sim22_2.0	17200.98	0.33 (0.05)	18.28 (14.41)	0.51 (0.05)	0.33 (0.05)	0.01 (0.06)	0.14 (0.19)
Sim22_1.5	16878.62	0.34 (0.05)	20.38 (14.57)	0.67 (0.05)	0.34 (0.05)	0.05 (0.08)	0.12 (0.15)
Sim22_1.0	17701.72	0.4 (0.05)	19.9 (12.93)	0.81 (0.04)	0.39 (0.05)	0.12 (0.09)	0.14 (0.14)
Sim22_0.5	18613.08	0.43 (0.05)	18.04 (10.92)	0.93 (0.03)	0.13 (0.03)	0.21 (0.1)	0.17 (0.1)
Sim22_0.25	20404.02	0.46 (0.05)	20.4 (11.63)	0.98 (0.01)	0 (0)	0.25 (0.11)	0.2 (0.11)
Sim22_bin	22847.06	0.42 (0.05)	26.22 (10.9)	0.49(0.07)	0.34(0.05)	0.2 (0.07)	0.15 (0.08)

IntScreen (given as mean) is the number of selected interactions by the corresponding screening method; IntSensiA (given as ‘sensitivity value (sd)’) is the sensitivity related to the availability of true interactions; VarsTotal (given as ‘mean (sd)’) is the number of total variables in the final model; MainSensi (given as ‘sensitivity value (sd)’) is the sensitivity related to the inclusion of true main effects; and IntSensi (given as ‘sensitivity value (sd)’) is the sensitivity related to the inclusion of true interactions. The rIPEC values (given as ‘mean (sd)’) are shown for CoxBoostM and the final model. The scenarios were repeated 50 times. Additional file 1: Figure S2 provides boxplots for further insights into the nature of the variability in rIPEC.

In Table 4 results of the scenarios with correlations are given. These scenarios are challenging, because random forests can have problems in distinguishing between correlation and interactions. The problem is dealt with extensively in [57]. There is a debate whether correlations are pointing to relevant associations or not (see [78-80]). Our focus here is on the effect of non-informative correlations on MainSensi and IntSensi. The results show that even small correlation values lead to decreased sensitivities compared to Sim22_1.0. Both, CoxBoost and random forest are negatively but not overly affected by correlations. However, with correlations ≥0.5 IntSensi and MainSensi decrease excessively. InterSensiA values of 0.15 and InterSensi values of about 0.05 suggest that most of this deterioration can be ascribed to CoxBoost and not to the random forests. The rIPEC does more or less reflect the tendency of reduced sensitivities. In summary, correlations do pose a problem for rsf-VIF-res, but mainly because of the inability of CoxBoost to select the true effects and not because of the random forests component. This is corroborated by the fact (data not shown) that true interactions were almost never replaced by interaction terms built by variables that correlate with variables in the true interactions.

**Table 4:** **Results of the simulation study for **rsf-VIF-res** in scenarios with correlated variables**

	**IntScreen**	**IntSensiA**	**VarsTotal**	**MainSensi**	**IntSensi**	**rIPEC**	
						**CoxBoostM**	**Final Model**
Sim22_corr01	19779.06	0.22 (0.04)	17.76 (13.25)	0.75 (0.04)	0.21 (0.04)	0.11 (0.07)	0.1 (0.11)
Sim22_corr03	17961.56	0.23 (0.04)	12.74 (12.85)	0.46 (0.05)	0.18 (0.04)	0.06 (0.07)	0.03 (0.08)
Sim22_corr05	15953.22	0.15 (0.04)	6.66 (8.6)	0.14 (0.03)	0.06 (0.02)	0.03 (0.06)	0 (0.03)
Sim22_corr07	14174.12	0.15 (0.04)	7.26 (8.49)	0.06 (0.02)	0.04 (0.02)	-0.03 (0.2)	0.01 (0.3)

IntScreen (given as mean) is the number of selected interactions by the corresponding screening method; IntSensiA (given as ‘sensitivity value (sd)’) is the sensitivity related to the availability of true interactions; VarsTotal (given as ‘mean (sd)’) is the number of total variables in the final model; MainSensi (given as ‘sensitivity value (sd)’) is the sensitivity related to the inclusion of true main effects; and IntSensi (given as ‘sensitivity value (sd)’) is the sensitivity related to the inclusion of true interactions. The rIPEC values (given as ‘mean (sd)’) are shown for CoxBoostM and the final model. The scenarios were repeated 50 times.

### Real data illustrations

#### Diffuse large-B-cell lymphoma data

In order to illustrate how rsf-VIF-res can be applied on real data, we first analyzed the well-known Rosenwald data [81]. This data set was used to link 7399 (‘lymphochip’ cDNA microarray) gene expression features of 240 patients with diffuse large-B-cell lymphoma (DLBCL) to the time of their death. DLBCL is an aggressive malignancy of mature B lymphocytes with a high rate of remissions. The objective of the Rosenwald study was to devise a molecular profile that accounts for the underlying heterogeneity, predicts survival and can be used for assessing the effect of the related therapies. Overall, 138 deaths were observed, with a five year overall survival of 48%. The 7399 features measured at baseline represent 4128 genes. An established clinical predictor, the International Prognostic Index (IPI - a combination of five clinical features), is available for *n*=222 patients, which will be considered for the analysis in the following, i.e. K={Indices(IPI)}. For further details and an overview with respect to various strategies for analyzing this and related data sets, we refer to [82].

**Figure 1:**
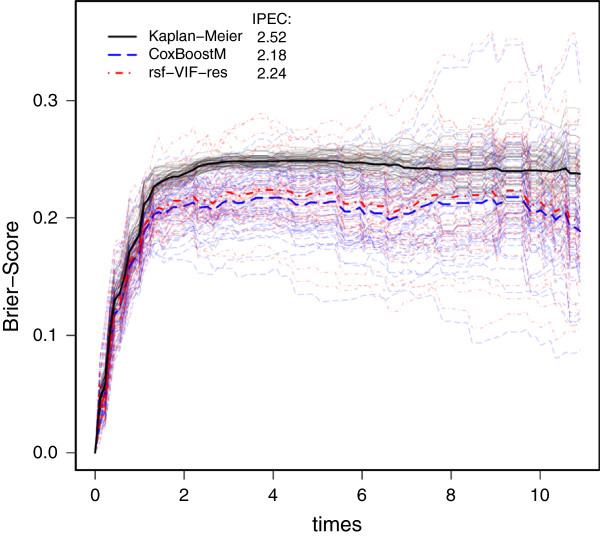
**Prediction error curves on the Rosenwald data.** Shown are the curves for the Kaplan-Meier estimates, CoxBoostM and rsf-VIF-res on all subsampled data. The bold curves are the aggregated curves over all subsamples. Additionally, rIPEC values are given.

We were interested in gaining new insights by incorporating interactions together with main effects. In almost all previous analyses of the Rosenwald data, at least four genes exhibited strong main effects. Our assumption was that there should be relevant interactions as well. Even though, it is frequently reasonable to assume complex and non-linear interactions, using cross product terms should be a first step in enriching the molecular profile. rsf-VIF-res was applied with *R*=10,000 and on 50 subsamples of the original data set. The prediction error curves of CoxBoostM and of rsf-VIF-res are given in Figure 1. The mean prediction errors (bold dashed lines) show that rsf-VIF-res performs slightly worse than CoxBoostM. Based on our assumption that there should be relevant interactions, the simulation study suggests that slight reduction of the prediction performance might still point to interaction effect sizes that are moderate, i.e., below the effect sizes of the main effects but not negligible (see Sim22_0.25).

The three main effects and gene-gene-interactions, given in Unigene cluster notation, related to the largest relative VIFs (in parentheses) are: 

- Hs.184298 (0.66), Hs.99741 (0.54), Hs.85769 (0.44) and

- Hs.76807:Hs.84298 (0.10), Hs.79428:Hs.193736 (0.08), Hs.20191:Hs.99597 (0.06)

**Figure 2:**
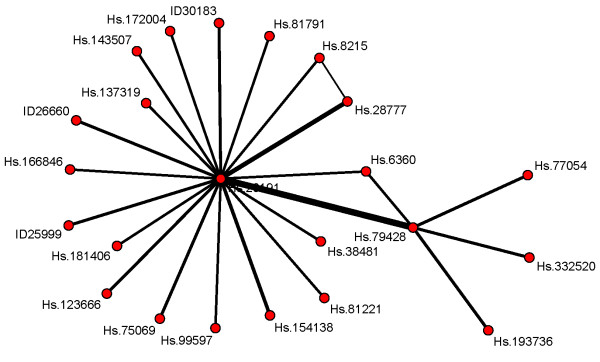
**Network graph for the Rosenwald data depicting the connections between those interaction that are found with relative VIF ≥3/50.** The thickness of an edge reflects the value of the corresponding VIF, i.e., the VIF value of an interaction term, the bolder the edge between the variables in that interaction term. For microarray features that do not correspond to a gene, the feature ID is given. Every other molecular feature is represented by the Unigene cluster notation.

The underlying genes of the interactions represent no relevant main effects for CoxBoostM, and the VIFs of these interactions are low. In order to increase certainty concerning the interactions, we manually increased the step size of the final model to 500. There, the same main effects are associated with slightly higher VIFs (0.72, 0.6, 0.52). The changes for the interactions are more interesting: two new interaction terms are among the interactions with the largest VIFs and the relative VIF values increased to 0.22, 0.16, and 0.14 for Hs.20191:Hs.79428, Hs.20191:Hs.28777, and Hs.76807:Hs.84298, respectively. From the considerable increase of relative VIFs, we concluded that these interactions might be more reliable. Figure 2 shows the connections between the genes in selected interaction terms with relative VIFs ≥3/50 (the bolder the edges, the higher the corresponding VIF values). Our observations in the simulation study (specifically Sim22_0.5) indicate that the most frequent interaction term Hs.20191:Hs.79428 could be relevant, although its frequency is moderate. However, mean model size increased drastically from 24 to 84 and led to an rIPEC value of 0, so, in order to corroborate our conclusion further, we went back to molecular biological information. From KEGG (Kyoto Encyclopedia of Genes and Genomes), we retrieved the pathways of the genes in the interaction term Hs.20191:Hs.79428. The proteins of these genes (SIAH and BNIP3) are elements in the pathway related to (mitochondrial) apoptosis [83,84]. In addition to that, they have a role in the cellular response to hypoxia [85,86]. The expression values of both genes lead us to the assumption that the corresponding proteins might interact complementarily: common down /up-regulation with respect to the apoptotic function and to hypoxial-induced reactions might have an impact on tumor genesis and growth (see also [87,88] for further evidence).

#### Neuroblastoma data

**Figure 3:**
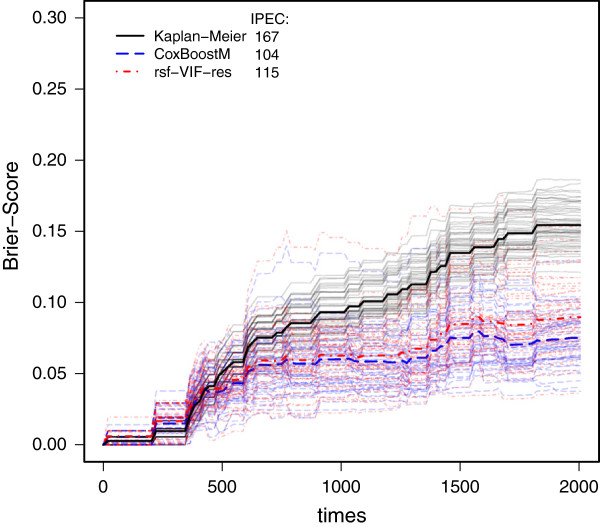
**Prediction error curves on the Neuroblastoma data.** Shown are the curves for the Kaplan-Meier estimates, CoxBoostM and rsf-VIF-res on all subsampled data. The bold curves are the aggregated curves over all subsamples. Additionally, rIPEC values are given.

A further real-world example is related to the microarray data set of Oberthuer et al [89]. It consists of *n*=276 patients suffering from neuroblastoma. Overall, 42 deaths were observed and the median survival time is 632 days. For each patient, *p*=9,986 microarray features are available, and we concentrate on the relationship between survival and these microarray features. The same parameter values as for the Rosenwald data are used but with no clinical covariates, i.e. K={}. The prediction error curves of CoxBoostM and of rsf-VIF-res are given in Figure 3. Again, the mean prediction errors (bold dashed lines) indicate that rsf-VIF-res performs slightly worse than CoxBoostM.

The three main effects and gene-gene-interactions with the largest relative VIFs are: 

- Hs.496658 (0.68), Hs.491494 (0.58), Hs.584827(0.54) and

- Hs.496658:Hs.148989 (0.28), Hs.496658:Hs.371249 (0.18), Hs.496658:Hs.532824 (0.18)

**Figure 4:**
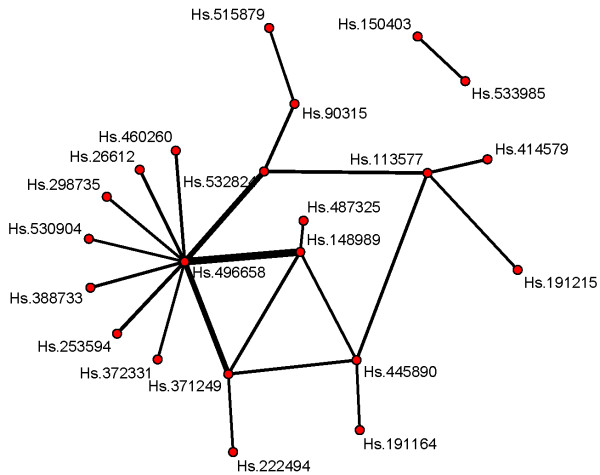
**Network graph for the Neuroblastoma data depicting the connections between those interaction that are found with relative VIF ≥3/50.** The thickness of an edge reflects the value of the corresponding VIF, i.e., the VIF value of an interaction term, the bolder the edge between the variables in that interaction term. The molecular features feature are represented by the Unigene cluster notation.

VIFs are higher than for the Rosenwald data. Based on the simulation study results, the VIFs might be considered large enough for indicating important interactions. Hs.496658 is the most relevant gene entity: it contributes the largest main effect VIF and is involved in interactions with the largest VIFs. The corresponding gene name is SLC25A5, and the product of this gene functions as a gated pore that translocates ADP from the mitochondrial matrix into the cytoplasm. Suppressed expression of this gene has been shown to induce apoptosis and inhibit tumor growth (see the corresponding entry in the database of NCBI). Figure 4 shows the connections between the genes from interactions with relative VIFs ≥3/50. The graph is more complex than Figure 2, which translates into an increased uncertainty with respect to the relevance of the interactions. For example, Hs.148989 is gene CGNL1, which encodes a protein that localizes to both adherens and tight cell-cell junctions and mediates junction assembly and maintenance (see the corresponding entry in the database of NCBI). There could be a real interaction between both genes (e.g., when cell-cell junctions break loose, apoptosis cannot be induced), but further biological validation would definitely be necessary.

## Discussion

From the results of the simulation study, we conclude that random forests can provide relevant interaction information. If the interaction is strong enough, the marginal effects of the underlying variables are at a level such that they are frequently selected as split variables in the random forest generation process. Further, the results indicate that disentangling information also is important for achieving good results. The reason behind this might be that variables associated to main effects can mask interactions in random forests, which affects the split variable selection process. Disentanglement of information specifically means to transform variables to be orthogonal to those with indices in (ℳ∪K). When the number of estimated main effects in CoxBoostM is too large (rule of thumb: more than about $\frac{1}{10}\cdot n$[90]), the corresponding regressions can be unreliable. In this case, we would recommend focusing on those main effects with the largest absolute coefficient estimates in CoxBoostM. Another possibility is to use the linear predictor $\text{lp}=\underset{i\in ℳ}{\sum }\beta_{i}x_{i}$ for the regression $x_{\text{jk}}=\alpha \cdot {\text{lp}}_{k}+\varepsilon_{\text{jk}},\;j\notin ℳ,k=1,\ldots ,n$. In both cases, the orthogonalization is imperfect and results (not shown) based on the latter variant indicated that sensitivities related to interactions are considerably lower than with the strategy for computing residuals proposed here. However, an alternative should be taken into consideration, when the number of cases is small (e.g., smaller than 50).

The scenarios with correlation and non-smooth interactions show that the pre-selection of interactions is less affected than the final CoxBoost model. For non-smooth interactions, this was expected due to the non-smooth nature of individual trees in random forests, but the effects on correlated data indicate that the pre-selection of interactions in rsf-VIF-res is quite robust. One further interpretation of the simulation study is that moderate variable inclusion frequency of an interaction term (e.g., 10*%*−30*%*) still could indicate an important interaction. The real data example showed that uncertainty related to the reliability of the findings can make it necessary to consider and contextualize as much information as possible. Specifically, increasing the step size of CoxBoost from its optimal value to 500 in the Rosenwald data was an attempt to reduce the uncertainty. Due to the considerable deterioration of prediction performance, the decision on the importance of the identified interactions was based on additional biological knowledge. There is no absolute threshold with respect to a decrease in prediction performance that makes the results definitely unreliable. The results showed that detection of true interaction and main effects can be accompanied by deteriorated or bad prediction performances due to the increase in false positives. It always depends on the subject-matter question whether a certain level of prediction performance is deemed necessary. If biology can help sorting out the true effects, concerns related to prediction performance even might be considered secondary.

The results showed that the number of pre-selected interactions *R* must be large enough (>>1000 in our data sets) for guaranteeing that the screening process is able to pre-select relevant effects. CoxBoost was frequently able to select the right variables out of ten thousands of variables. This is a feature of many other (*L*_1_-) regularized regression techniques such as the LASSO (see also [91,92]), which (under sparsity assumptions) also are consistent for variable selection, even when the number of variables *p* is as large as exp(*n*^*α*^) for some 0<*α*<1 [93]. Empirically determining an optimal *R* is nevertheless difficult. This issue certainly needs further scientific investigations.

## Limitations

Due to the focus of this paper and the limited space, our study has several limitations. First, only two-way interactions were considered in the interaction screening process. In real-world data, all kind of multifactor and non-linear interactions can be expected. Second, the simulation scenarios are limited in their scope, because we focused on one critical issue: the effects of the building blocks when interactions are built from variables that do not represent main effects. Although, we also investigated simulations scenarios with correlations, further investigations of informative correlations and more complex correlations structures are relevant.

Third, the real-data applications showed that the strategies cannot be used in an automatic way. Decisions related to the choice of some parameter values (e.g., number of subsamples *S* or indices of unpenalized variables ), interpretation of the results, and further processing of these results have to be based on subject-matter knowledge and the specific application. Such requirements could discourage a user from using rsf-VIF-res. Nevertheless, it should be clear that assessing the necessity of considering interactions is not trivial, even for the simplest case of gene-gene interactions and therefore informed decisions are crucial.

Fourth, there are open questions such as the specific value for *R* or alternatives to the building blocks presented in the paper. There are several routes for extending our proposal or replacing components in it. Fifth, we only considered proportional hazard models and simulated data from such models. It is important to consider departures from the related assumptions in future studies, for example by considering time-dependent effects. Finally, the real data examples only considered microarray data. Recent sequencing approaches, such as the RNA-Seq technology, are gaining more and more ground and should be targeted as well.

## Conclusion

Our aim in this study was to build a strategy for incorporating two-way interactions into multivariate risk prediction models that are built on high-dimensional molecular data. When it is either not feasible or computationally too expensive to consider all possible interactions, screening is necessary in case of no a priori knowledge. We presented three important building blocks for such a screening strategy: subsampling, random forests, and orthogonalization of the data, and concluded that all building blocks are important. Our decision for using random forests for screening interactions has one main reason: the promise of random forests to capture various kinds of relevant interaction structures. CoxBoost was used, because it usually produces sparse risk prediction models. We assumed that a combination of these two approaches could be fruitful due to their complementary character. However, components can be separately replaced by other ones, for example random forests by multifactor-dimensionality reduction, and such flexibility seems necessary, because no specific combination of building blocks will perform well on every kind of data.

The results show that screening interactions through random forests is feasible and useful, when one is interested in finding relevant two-way interactions. Effect sizes of the interactions should be large enough in order to guarantee useful results. When the underlying variables do not represent main effects, sensitivities related to variable and interaction selection are moderate (≤40*%*). The results of the simulation study indicates that making all variables orthogonal to those with indices in (ℳ∪K) could enable random forests to pre-select relevant interaction effects even in the absence of strong marginal components.

The real data applications showed that not only pre-processing and a combination of different tools are important for interaction detection but also an intelligent post-processing. Our final conclusion is that in addition to focusing on establishing new methods, it is important to make full use of existing ones.

## Competing interests

The authors declare that they have no competing interests.

## Authors’ contributions

MS and HB developed the approach and the design. IH implemented the pseudo-algorithm, conducted the simulation study, applied the approach to the real data, and contributed to design decisions. The work of IH fulfills part of the requirements of her PhD. MS supervised the implementation process and wrote most of the manuscript. All authors read and approved the final manuscript.

## Supplementary Material

Additional file 1 **Supplementary tables and figures.** One table with respect to different values of R in the simulation study. Two boxplots for further insights into the nature of the variability in rIPEC. Click here for file
