# Supplementary material for: Combining techniques for screening and evaluating interaction terms on high-dimensional time-to-event data
Source: BMC Bioinformatics. 2014 Feb 26;15:58. doi: 10.1186/1471-2105-15-58 (PMC3945780; doi:10.1186/1471-2105-15-58)

Table S1 Results of rsf-VIF-res in scenarios Sim42 and Sim22_1.0 for R=10000 and R=1000

Scenario	R	IntScreen	IntSensiA	VarsTotal	MainSensi	IntSensi	rIPEC	
							CoxBoostM	Final Model
Sim22_1.0	10000	15231.62	0.3	16.04 (14.72)	0.29 (0.0454)	0.3 (0.0458)	0 (0.059)	0.103 (0.162)
	1000	1597.9	0.02	11.58 (12.9)	0.31 (0.0462)	0.02 (0.014)	-0.004 (0.063)	-0.007 (0.087)
Sim42	10000	25740.32	0.97	32.02 (6.56)	0.845 (0.0362)	0.97 (0.0171)	0.123 (0.122)	0.403 (0.116)
	1000	2622.68	0.81	21.96 (8.29)	0.85 (0.0357)	0.81 (0.0392)	0.123 (0.121)	0.39 (0.193)

Results are given in the form 'mean (sd)'. IntScreen is the number of selected interactions by the corresponding screening method, VarsTotal is the number of total variables in the final model, MainSensi is the sensitivity related to the inclusion of true main effects, IntSensiA is the sensitivity related to the availability of true interactions, and IntSensi is the sensitivity related to the inclusion of true interactions. The rIPEC values are given for the initial CoxBoost (CoxBoostM) and the final model.

Figure S1 Boxplots of rIPEC values in scenario Sim42 and Sim22_1.0 for CoxBoostM and all strategies

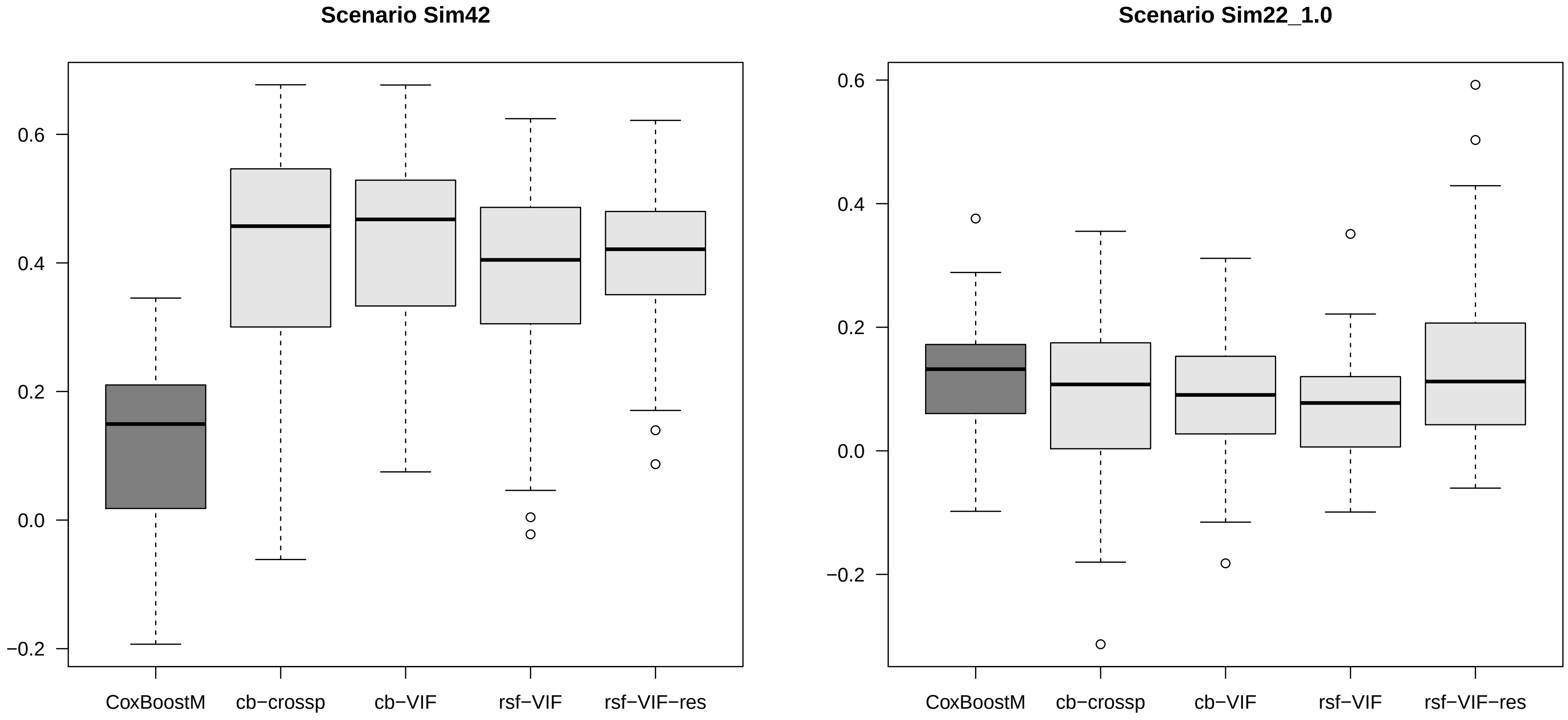

Figure S2 Boxplots of rIPEC values for CoxBoostM and rsf-VIF-res in scenarios Sim22__0.25-Sim22__2.5

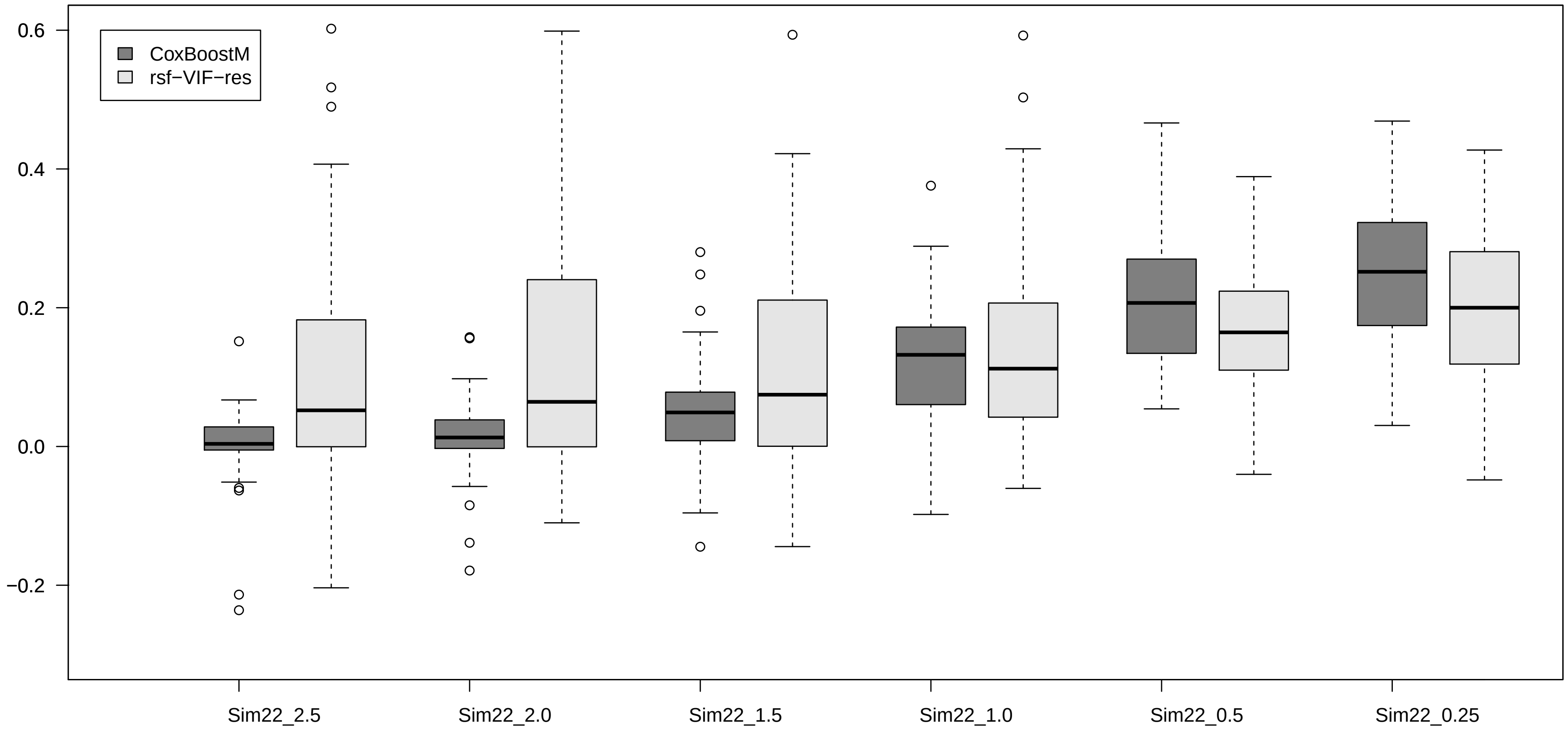

Supplement: Additional file 1 — Supplementary tables and figures. One table with respect to different values of R in the simulation study. Two boxplots for further insights into the nature of the variability in rIPEC. [file 1471-2105-15-58-S1.pdf]
